# Understanding biochemistry: basic aspects of statistics for life sciences

**DOI:** 10.1042/EBC20220211

**Published:** 2023-10-25

**Authors:** Donald Reid

**Affiliations:** University of Glasgow, School of Biodiversity, One Health and Veterinary Medicine, Room 332, Sir James Black Building, University of Glasgow, Glasgow G12 8QQ, U.K.

**Keywords:** analysis, biostatistics, general linear models, statistics

## Abstract

If the biological world is one thing it is variable. As scientists we seek to measure, quantify and explain the causes of this variation. The approach we take to this is remarkably similar whether our research is exploring global temperature, blood pressure, cancer incidence or enzyme kinetics. This approach involves defining clear research questions and applying statistical methods to answer them robustly. This article will introduce a practical example that will be used throughout, specifically whether genetic variation can explain variation in coffee consumption. We assume little experience with statistics and walk through the statistical approach that biologists can use, firstly by describing our data with summary statistics and then by using statistical tests to help arrive at answers to our research question. A General Linear Model (GLM) approach will be used as this is what many common statistical tests are. We explore how to visualise and report results, while checking the assumptions of our analysis. The better we can understand and apply statistics to biological problems, the better we can communicate results and future research to others. The popular statistical programming language R will be used throughout.

## Aim

The aim of this article is to introduce you to basic aspects of statistics that you can utilise as a biologist. We will introduce a practical example that will be used throughout to explore how we can use statistics to answer biological research questions. This is not the only method approach to data analysis, but it is a common one that we believe will be helpful for you. This guide is intended as an introduction that will make other data analysis resources accessible to you as you grow in your learning. The data and annotated R script are available as supplementary material linked at the end of the article. You can use this to follow along and repeat any analysis presented here for yourself.

## Our research question

Genetic variation is a core part of evolutionary biology, and its consequences are commonly explored throughout biochemistry research. The TAS2R38 gene encodes a protein called the phenylthiocarbamide (PTC) receptor, which is found on the surface of taste receptors on the tongue. If you have certain alleles of the TAS2R38 gene then you will be able to taste ‘bitter’ thiourea-containing compounds such as PTC. The taster allele is ‘T’ and the non-taster allele is ‘t’ to give the following possible taster genotypes: TT (strong tasters), Tt (weak tasters) and tt (non-tasters). Therefore, we may be interested to explore whether variation in the consumption of ‘bitter’ food or drink is explained by variation in the TAS2R38 genotype, e.g. coffee or brassica vegetable consumption (both considered bitter). Let’s write that research question so we are clear what we are investigating:

Q. Do different TAS2R38 genotypes lead to differences in coffee consumption?

We can rephrase this question in a clearer way for our statistical approach.

Q. Can variation in coffee consumption be explained by TAS2R38 genotype?

We’ll utilise this research question throughout this article to show how we as biologists can apply statistical tools to answer our research questions.

## Statistical software

Our data analysis to answer a research question will almost always be carried out with software on a computer. For this article, R has been used and common commands stated when used (1). You do not need to be familiar with R to utilise this article. Other popular statistical software will give the same results in a similar format as you see here. Therefore, you can use this guide to facilitate your analysis and interpret results regardless of your preferred software.

R is a free, open-source programming language that is commonly used within biostatistics and beyond for data wrangling, visualisation and analysis. It has a great community with many forums, resources and guides to support biologists in their learning and application. The terms used in this article will hopefully make it easier for you to search these resources online. We would recommend using R within the popular R Studio integrated development environment (2), which makes it much easier to use R (3).

Link for R: https://www.r-project.org/

Link for R Studio: https://posit.co/products/open-source/rstudio/

## Types of variables

**Figure 1:**
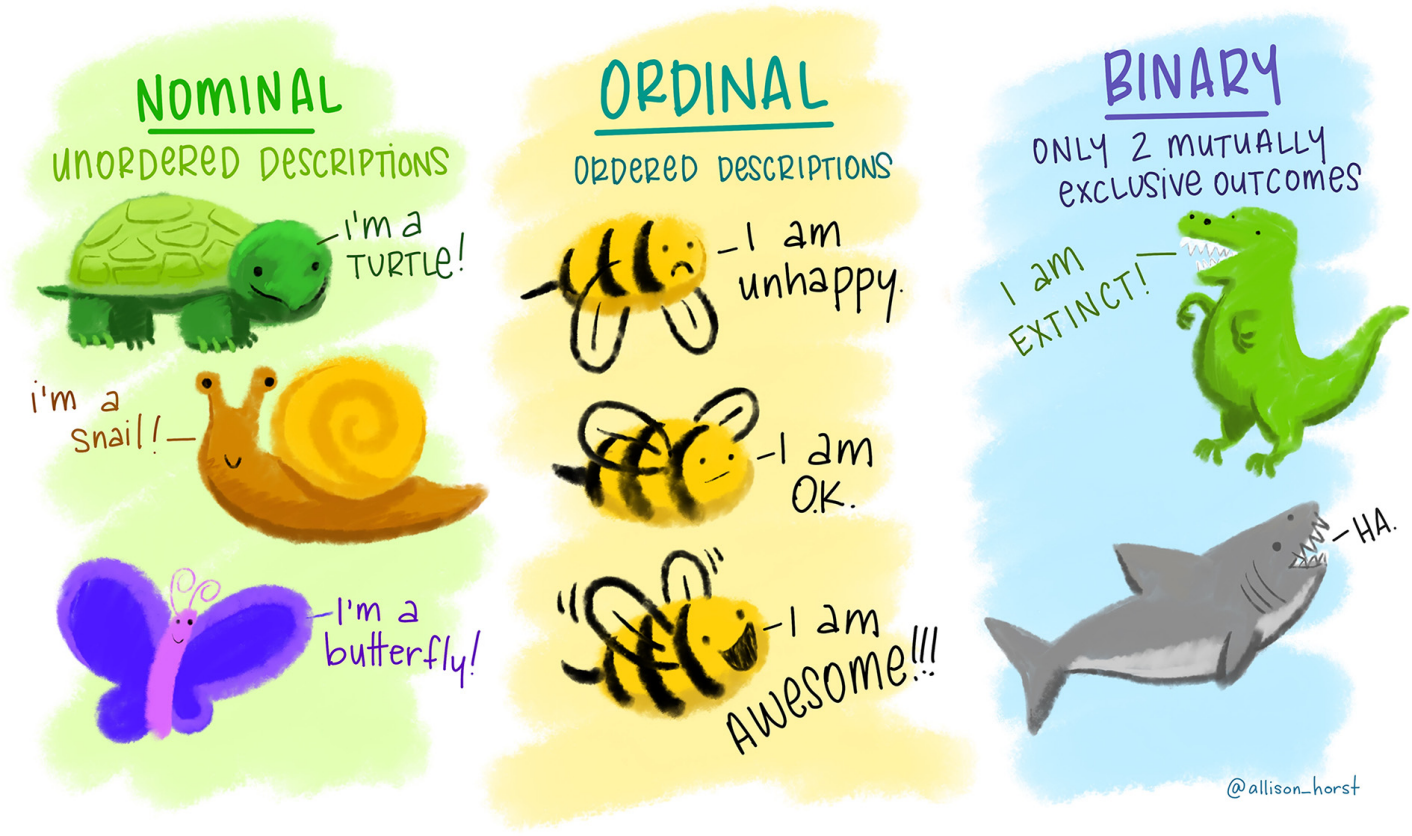
Ordinal, nominal and binary factor (categorical) variables Binary factor variables are a special case which may be consider either ordinal or nominal depending on the variable. Artwork by @allison_horst.

A variable is any characteristic that can be measured that varies. However not all these variables vary in the same way, so it is important to consider and classify different types of variable. For example, the TAS2R38 genotype varies between individuals as either TT, Tt or tt. Each person that we could measure will belong to one of these genotype categories and this is why we would classify genotype as a categorical variable, also called factor variable. Factor variables can be further classified by whether their groups can be ordered or not. If the groups, or levels, with a factor variable can be ordered (e.g. low/medium/high) then the factor is ordinal, if not (e.g. yes/no) then the factor is nominal. Figure 1 shows further examples.

**Figure 2:**
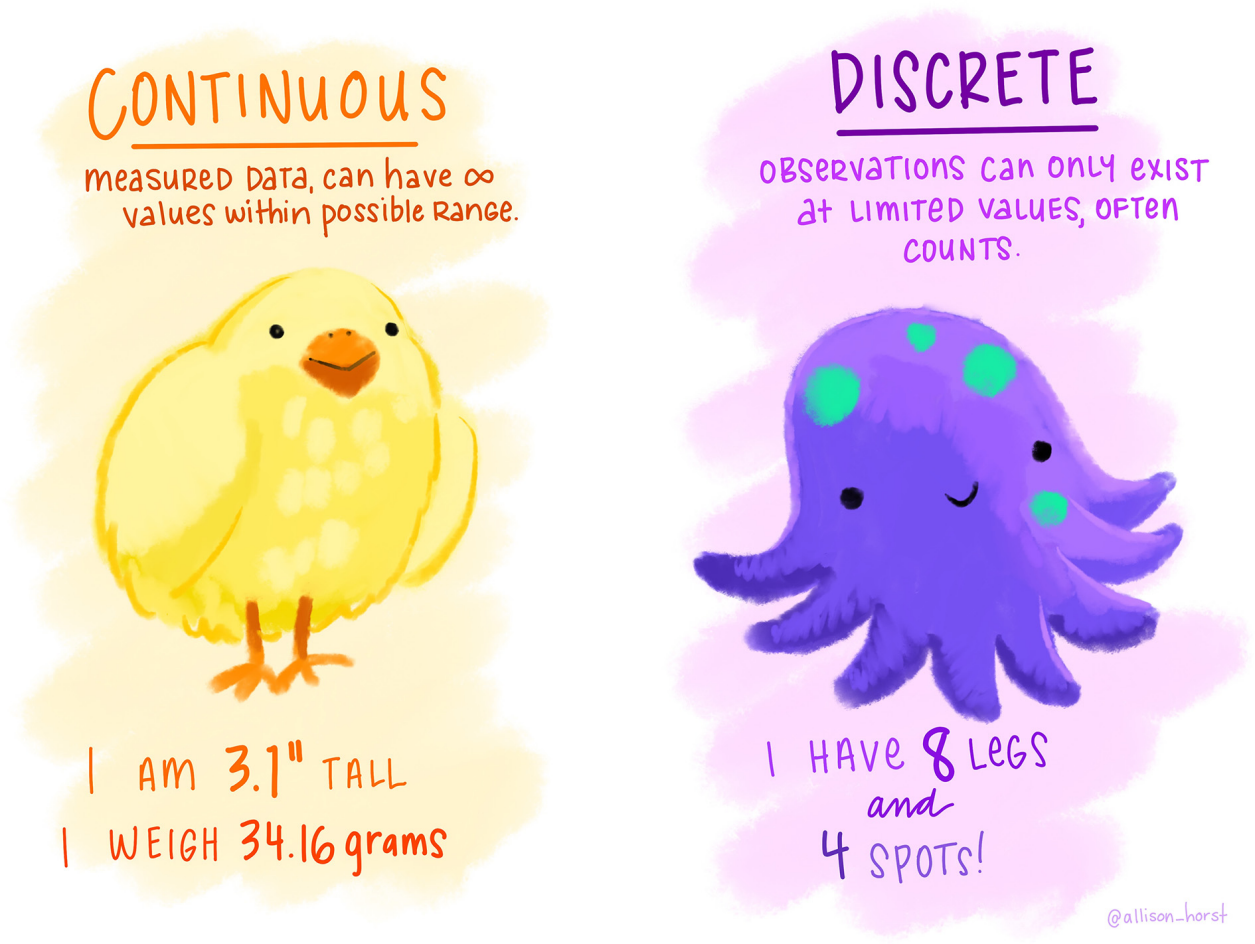
Continuous and discrete variables Artwork by @allison_horst.

Other variables to not fall into groups but are measured numerically. These are called numerical variables and often described as quantitative. For example, weekly coffee consumption would be measured in number of cups and be classified as a numerical variable. If this was measured as an integer, i.e. whole number of cups, it would be classified as discrete numerical. Some numerical variables are measured with more precision, on a continuous scale and classified as continuous numerical, e.g. temperature measured in degrees Celsius of 23.4°C. Figure 2 shows further examples.

You can check how R has classified your variables using the following command:

str(mydata)

You will need to replace ‘mydata’ with the actual name of your dataset.

**Figure 3:**
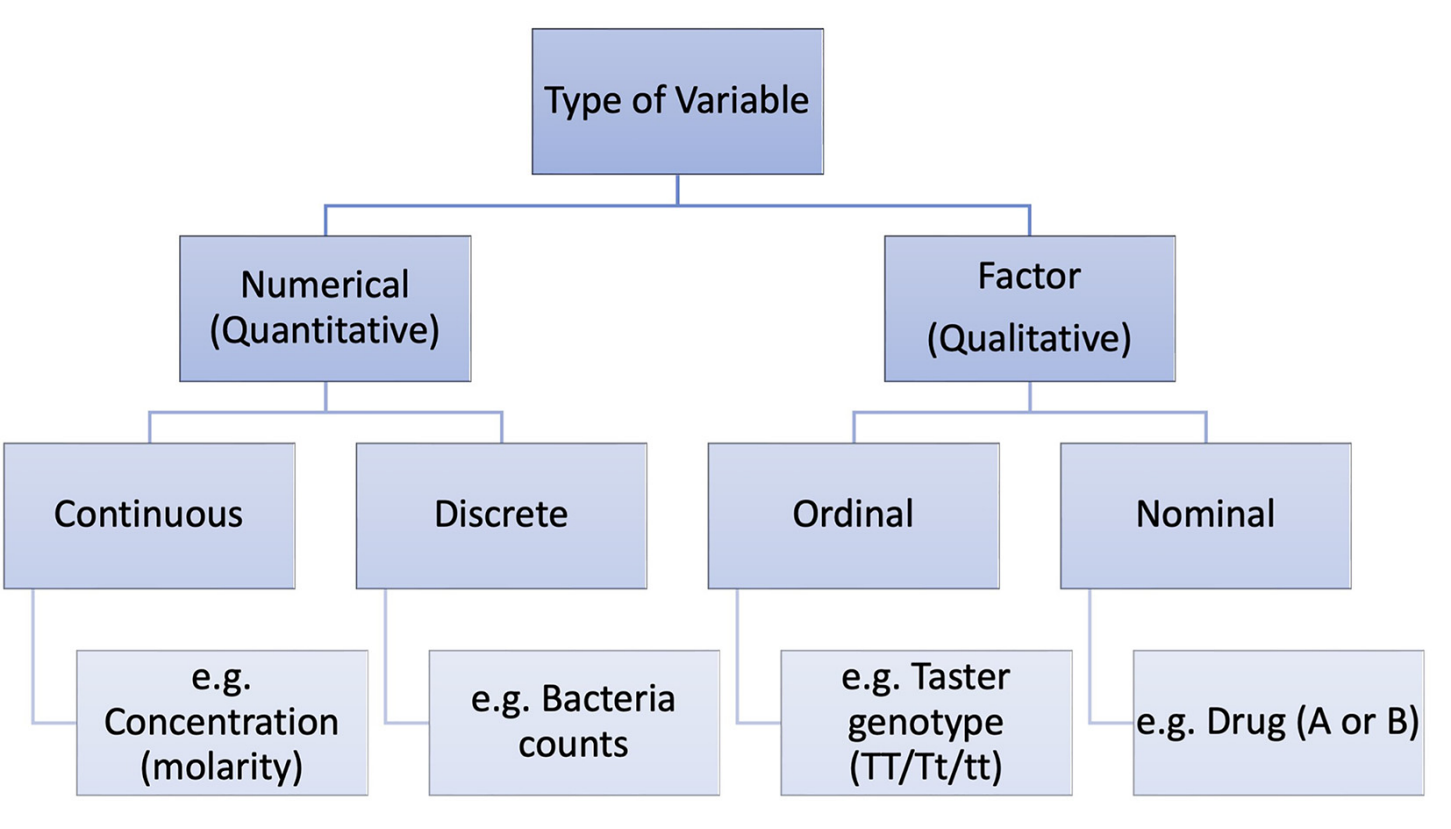
Classification of types of variables

Knowing which types of variables are in your research question will help you with your statistical analysis and interpretation of results. A summary of how we can classify variables is show in Figure 3.

## Descriptive statistics

It is key to be able to describe and quantify our variables. We can do this by obtaining summary statistics for our variables, which are much more accessible than raw data. This helps us understand and communicate patterns within our variables. Our variables can be described in both measures of centrality and variation.

You can obtain summary statistics in R using the following command:

summary(mydata)

You will need to replace ‘mydata’ with the actual name of your dataset. The dataset used to answer out question is called ‘PTC’ after phenylthiocarbamide, a thiourea-containing compound. The T allele confers the ability to taste PTC, and people who carry no T alleles and only t alleles cannot taste PTC.

**Figure 4:**
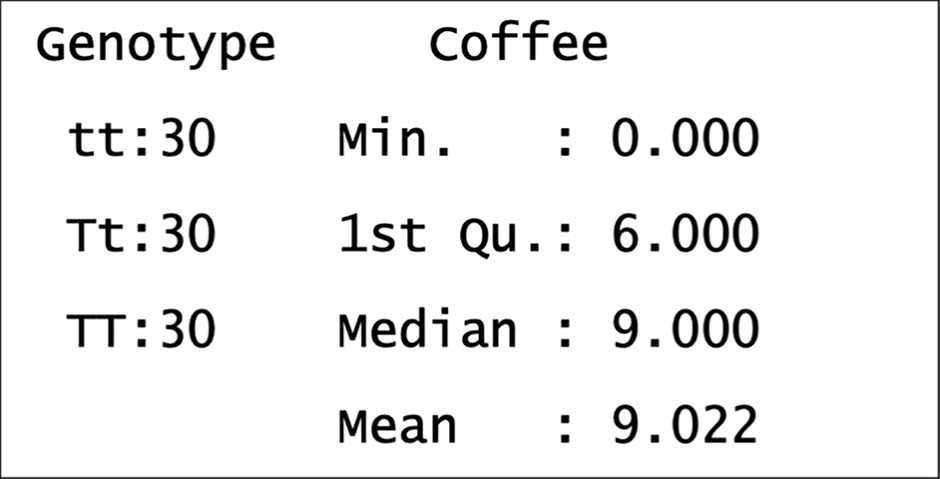
Summary statistics of our dataset

In Figure 4 you can see summary statistics from this dataset. For factor variables, such as genotype, the number of observations within each group (or level) is stated. This gives us an opportunity to understand and critique our dataset, e.g. each genotype group has 30 individuals so the dataset is well balanced. The sample size is 90 which is relatively small to measure behavioural consumptions in humans due to TAS2R38 taster genotype. This sample size could be considered large if the observations were of patients with a very rare condition or endangered animals, so the biological context may influence our critique. We desire our sample to be representative, so that we can make inferences about wider populations. One way to do this is to increase our sample size and to sample a broad spectrum of participants, e.g. avoid sampling related individuals or solely from a village renowned for its coffee consumption.

For numerical variables, such as weekly coffee consumption, we see in Figure 4 the minimum and maximum coffee consumption (cups per week) and other measures we will focus on soon e.g. 1st and 3rd quartiles. There are individuals who drink no coffee, as we would expect in the general population. The maximum coffee consumption is 21 cups per week, and this is perhaps lower than expected. This is not necessarily a problem but may make us sceptical of how representative this dataset is of the general population. This is one benefit of summary statistics, it allows us to understand and critique our data. This may help us appreciate limitations or caveat our conclusions.

## Measures of centrality

**Figure 5:**
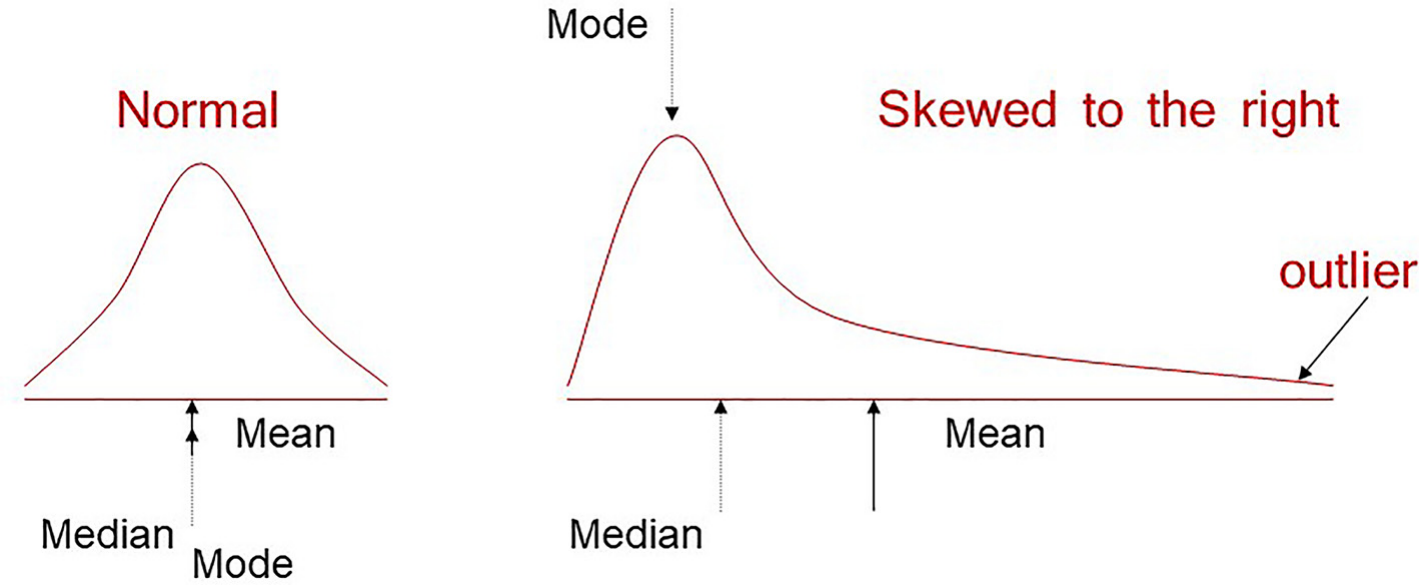
A normal and right-skewed distribution annotated with mean, median and mode

As the name suggests, measures of centrality seek to describe the mid-point of our numerical variable. The most commonly used is the mean, calculated as the sum of all observations divided by the sample size. The median (middle value) and mode (most common value) are other measure of centrality. When our variable is normally distributed (a bell-shaped curve also known as Gaussian), all three measures coincide but when our data are skewed these measures take different values (Figure 5). This is why it can be helpful to report more than the mean when our data are skewed. For example income data is routinely reported in median terms to be more representative of a mid-point salary as the mean is pulled up by a few extremely high-income earners.

## Measures of variation

**Figure 6:**
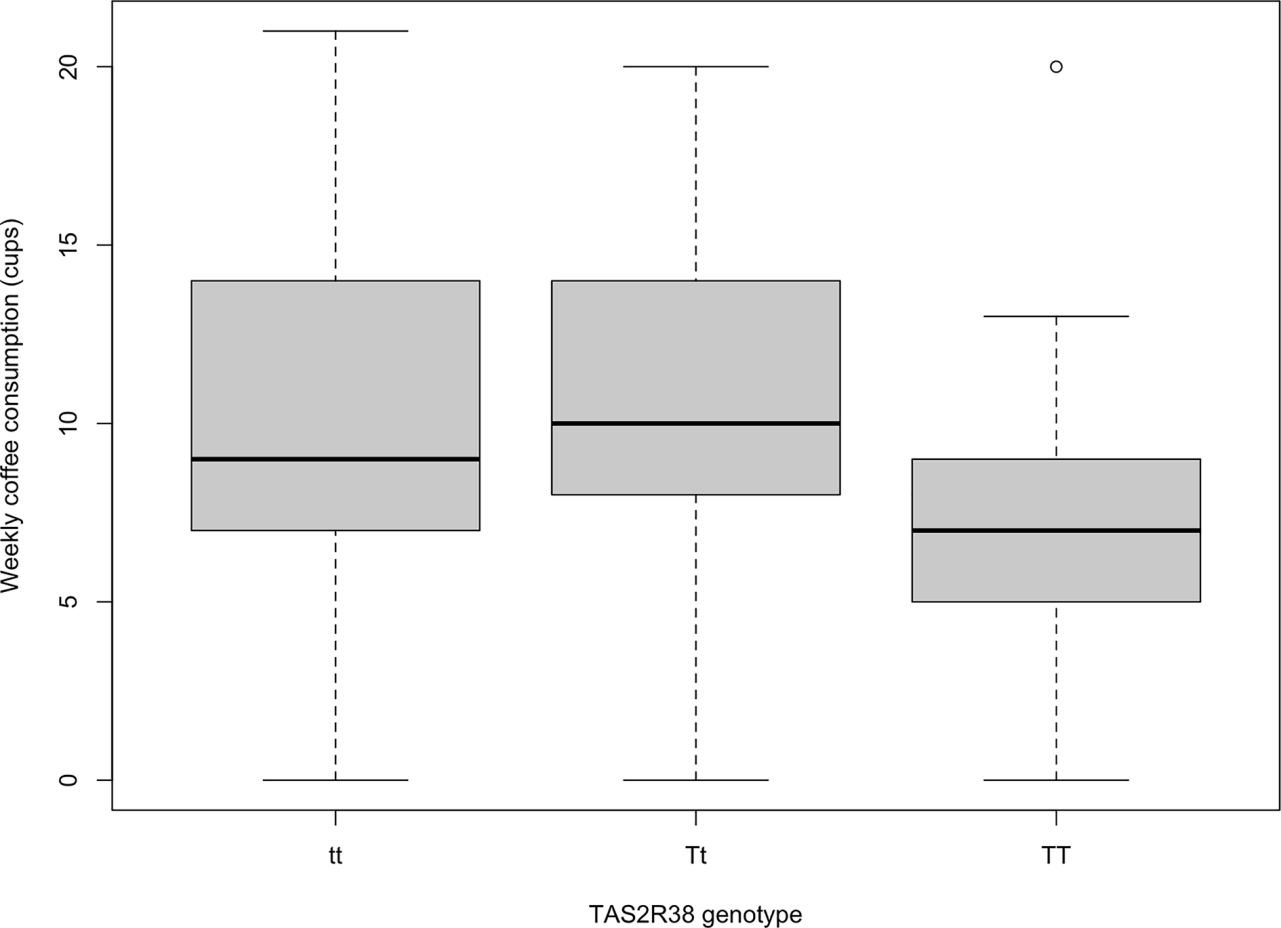
Boxplot of taster genotype and weekly coffee consumption (cups per week) tt genotype represents non-tasters, Tt weak tatsers and TT strong tasters for thiourea-containing compounds

Measures of variation help us appreciate the spread of our data. We may have two samples with the same mean that differ in the spread of observations, which is key to quantify and communicate. A simple measure is the range, which is the difference between the lowest and highest values of a variable. This is problematic as it is skewed by outliers and only informs us about the extremes of the variable, not the spread of our data. To address this the interquartile range (IQR) can be used where the sample size is divided into four quartiles, each including 25% of observations, and is calculated by the difference between the 25th and 75th percentile. You may be more familiar with the Interquartile Range when visually represented as the box of a boxplot. Figure 6 shows the boxplot for our research question.

**Figure 7:**
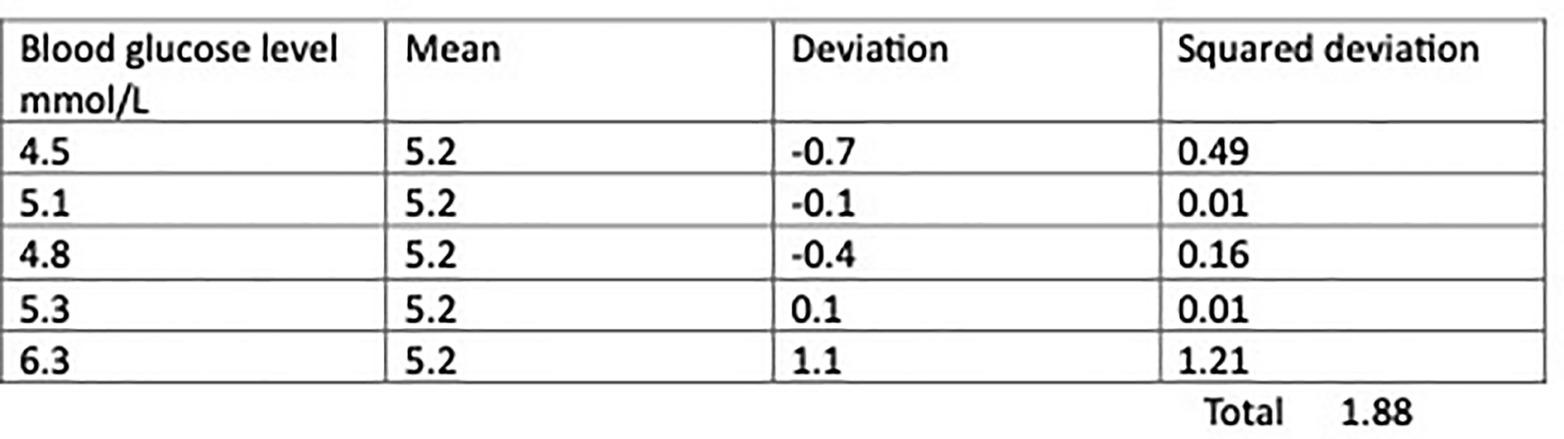
Table showing how sums of squares are calculated from deviations of datapoints from a common mean

To quantify variation, ideally we should incorporate information about the spread of every datapoint from a measure of centrality. If we had measures of blood glucose levels from five patients, we could calculate each of their deviation from a common mean (Figure 6). If we tried to combine these deviations to quantify variation in a simple total, the sum would be zero but if we square the deviations their sum will be a positive number that quantifies variation. This is known as Sum of Squares (SS) and is the measure of variation that many common statistical tests use in their calculations. Although each squared deviation no longer represents the exact spread of observations from the mean, they still reflect how far each point is from the mean, in the sense that the largest deviations correspond to the largest squared deviations (Figure 7).

Sums of Squares can also be divided by the sample size minus 1 to calculate variance. The standard deviation (SD) is the square root of the variance. Standard deviation is commonly reported to complement mean values as a measure of variation and incorporates the spread of all the data in its calculation.

**Figure 8:**
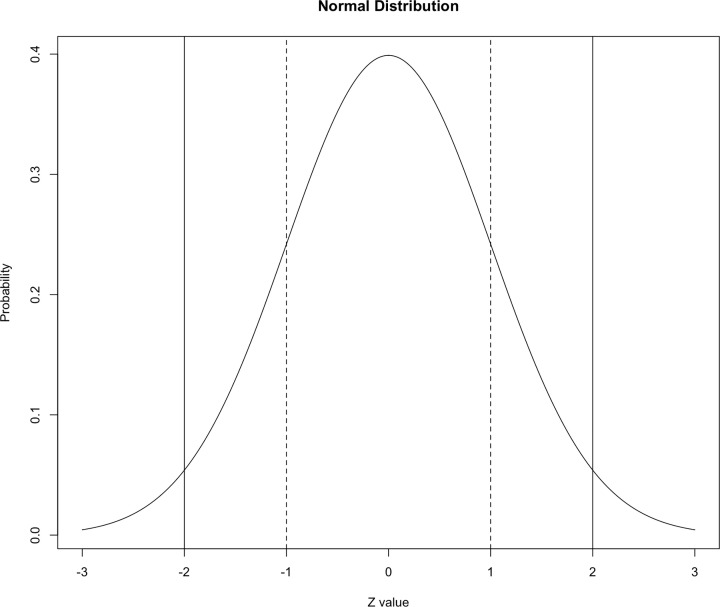
Density plot of a normal distribution The *Z*-value (*Z*-score) is the number of standard deviations a value departs from the mean, so the mean has a value of zero. Vertical dashed lines highlight one standard deviation above and below the mean. Vertical solid lines highlight two standard deviations above and below the mean.

Figure 8 shows how standard deviations relate to the mean of a normal distribution (Gaussian, bell-shaped curve). In this distribution 68.2% of the values are within one standard deviation of the mean (between vertical dashed lines), 95.4% of the values are within two standard deviations of the mean (between vertical solid lines) and 99.7% of the values are within three standard deviations of the mean. This knowledge is helpful when mean ± SD are reported in scientific papers as, although we may not have access to the raw data, we can expect the vast majority of observations to be within 2 SD of the mean. If we were replicating a study, it is helpful to know broadly what observations and variation we would expect to observe, and potentially very biologically interesting if we were observing many values outside this.

## Data visualisation

Measures of centrality and variation provide us with values to represent our variables but these summary statistics can hide patterns which can only be revealed by visualising our data. Exploring your data visually also allows comparison of variables, detection of data entry errors and identification of unusual values (outliers). Which plot we choose will depend on the variables we wish to visualise. For example, the relationship between two numerical variables can be visualised with a scatterplot while the relationship between a numerical and factor variable can be visualised with a boxplot. Factor versus factor comparisons are contrasting counts across groups and can be visualised by a barplot showing totals. The scope and effectiveness of data visualisation coding in R is huge and, although we will give you a little insight here it is beyond the scope of this paper. There are many great resources for visualising you data in R, including the R-Graph Gallery (https://r-graph-gallery.com/) and R-Graphics Cookbook (https://r-graphics.org/).

## Distributions

In Figure 5, there are examples of a normal (Gaussian) distribution and another distribution that is skewed to the right. This helped to demonstrate that measures of centrality and variation can inform us about the distribution of our data. Our data could show really any distribution and this is especially true in biology. It is not necessarily a problem if our data are not normally distributed, but it is important to appreciate what our distribution is because that may influence our approach to analysis. In certain biological research areas, some distributions are more commonly observed than others and as a result you may find yourself becoming more familiar with particular distributions. Histograms and density plots help us to visualise the distribution of our variables, and can often be a clue to whether we may be sampling from normal distribution(s) or not. Histograms group our observations into ‘bins’ so we can visualise where we are observing similar values. Density plots also represent the distribution of a variable and appear as a smoothed version of the histogram (e.g. Figure 8).

**Figure 9:**
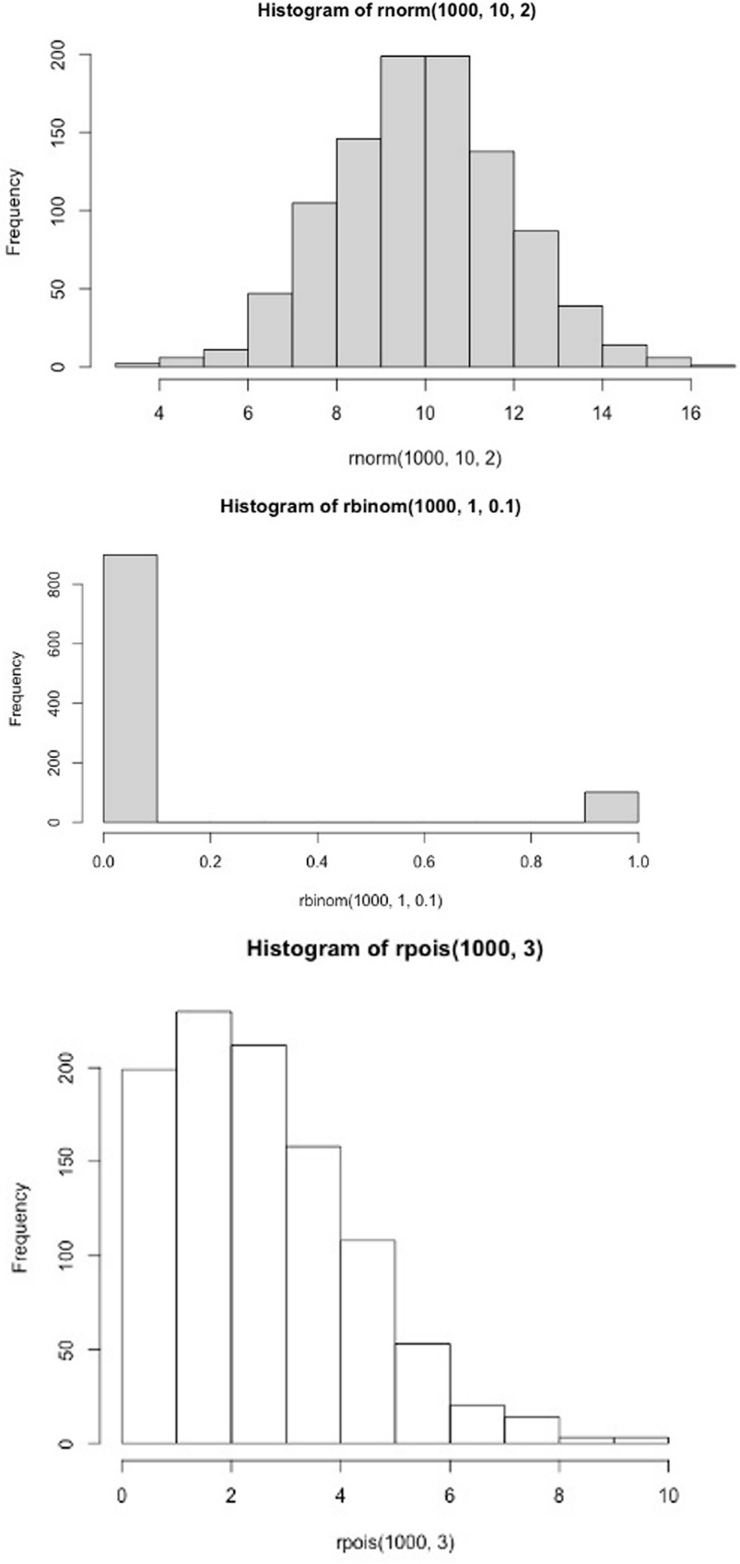
Histograms of 1000 observations sampled from three distributions commonly observed in biology with their parameters stated above each plot First a normal histogram with mean of 10 and standard deviation of 2, second a Poisson distribution with mean of 3 and finally a Bernoulli (binary) distribution of 0.1 probability of observing a 1 instead of a zero.

Some common distributions can be seen in Figure 9 and the arguments, or parameters, that define them. A normal distribution is defined by its mean and standard deviation. Other distributions are defined by different arguments, which is important to appreciate if undertake more advanced statistics. Statistical description by mean ± SD suits a normal distribution more than a Poisson distribution, where the data are skewed to the right and a subtraction of 2xSD would lead to negative values; however, negative values are not possible with a Poisson distribution. As biologists trying to make sense of our data, it is critical to visualise the distribution of our variables. Seeing our distributions reveals clues, that will inform our approach to understanding and analysing our data.

## Probability

There are whole books and courses dedicated to probability and its theory, but this is a brief introduction to probability focused on applying statistics for biologists. Probability quantifies anticipation of outcome and is usually represented by the letter p or P. We all have experience of probability and expectation of an outcome. When a coin is flipped, the expectation is that it will land on Heads half of the time, i.e. that P(H) = 0.5. If we were sampling from the normal distribution in Figure 8, it would be more probable to observe values around 10 then values around 5. It would not be very probable to observe a value of 1, but not impossible. The density plot in Figure 7 shows probability density on the *y*-axis. Where there is more area under the curve there is a higher probability of observing that value. We would not expect to regularly be observing values more than two standard deviations away from the mean in a normal distribution [1–7].

Probabilities are bounded between 0 and 1, with lower *P*-values representing a lower chance of occurrence. Strictly *P*-values are never zero, but they can be very small. When we perform statistical tests we obtain a p-value, and we will walk through this process with a General Linear Model later. Common statistical tests can be thought of as a purposeful calculation (sometimes a very long calculation) that arrives at a test statistic. You may be familiar with obtaining a Chi-squared value from a Chi-Squared test, a *t-*value from a *t*-test or an F statistic from a General Linear Model. These are all test statistics and come with a corresponding *P*-value. This *P*-value represents the probability of the statistic being that extreme or more if the null hypothesis is true (assuming assumptions of the test have been met). There is debate about what *P*-value is sufficiently low to be considered a threshold value, but you may have encountered the most commonly used of *P*<0.05. We’ll return to this concept later in the ‘Hypothesis Testing’ section.

## Inference statistics

Inference statistics is the process of using data analysis and hypothesis testing on our sample of data to estimate parameters in a population. A parameter is a property of the population, and a statistic is a property of the sample. We may calculate how much more coffee the non-taster group for bitter taste perception drinks than strong tasters from our sample and then make inferences about that of the general population. We are no longer describing our data but analysing it to answer our research question. This can often be achieved with use of General Linear Models (GLMs), which is a collective term for multiple tests including regression and ANOVA (analysis of variance). They are called linear models because they describe the relationship between our variables with linear equations. If you are interested in why many common tests are GLMs you can read this excellent article by Jonas Kristoffer Lindeløv: https://lindeloev.github.io/tests-as-linear/.

## Hypothesis testing

As scientists we seek to explain possible causes of observed variation. For example, whether variation in weekly coffee consumption is due to differences in taster genotype. A research question can usually be expressed as a pair of hypotheses, for example:

Null (H_0_): taster genotype DOES NOT account for variation in weekly coffee consumption
Alternate (H_a_): taster genotype CAN account for variation in weekly coffee consumption

If we measure 90 adults’ weekly coffee consumption and also identify their TAS2R38 taster genotype, we can see which of these two mutually exclusive hypotheses is supported by our data. When we run a statistical test, we calculate a test statistic and associated p-value that allows us to choose between these hypotheses. This is hypothesis testing. For good, or bad, the widely agreed threshold *P*-value for choosing between these hypotheses is 0.05. If *P*<0.05, we reject the null hypothesis and if *P*≥0.05 we do not reject the null hypothesis. This helps us arrive at a conclusion to our research question, e.g. TAS2R38 genotype does influence coffee consumption. Remember that this conclusion is based on our data, which often may have bias or nuance due to experimental design and sample size. This article is not focused on experimental design, but it is essential to consider before undertaking your data collection to ensure your data is as representative as it can be of the population you wish to make conclusions about.

## General linear model mechanics (as this is what most common tests are)

**Figure 10:**
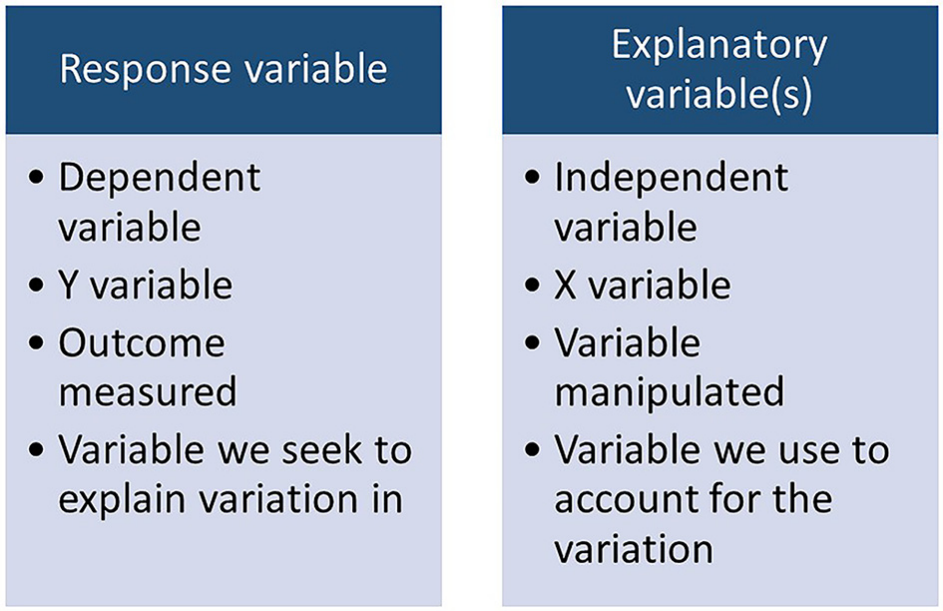
Synonyms and definitions of response and explanatory variables for general linear models

A General Linear Model describes the relationship between your response and explanatory variable. You can think of your response variable as being the outcome or focus of your research question. The explanatory variable is what you expect to explain your outcome or results. We will continue to refer to our variables as response and explanatory, but it is important to be aware of the synonyms sometimes used to describe these terms, shown in Figure 10. We may have multiple explanatory variables or only one, but the process is the same. It does not test for causality, and we should always place our findings in the biological context to identify if there is a plausible causal mechanism. There are plenty of examples demonstrating that correlation does not equal causation, including from Tyler Vigen (https://www.tylervigen.com/spurious-correlations).

**Figure 11:**
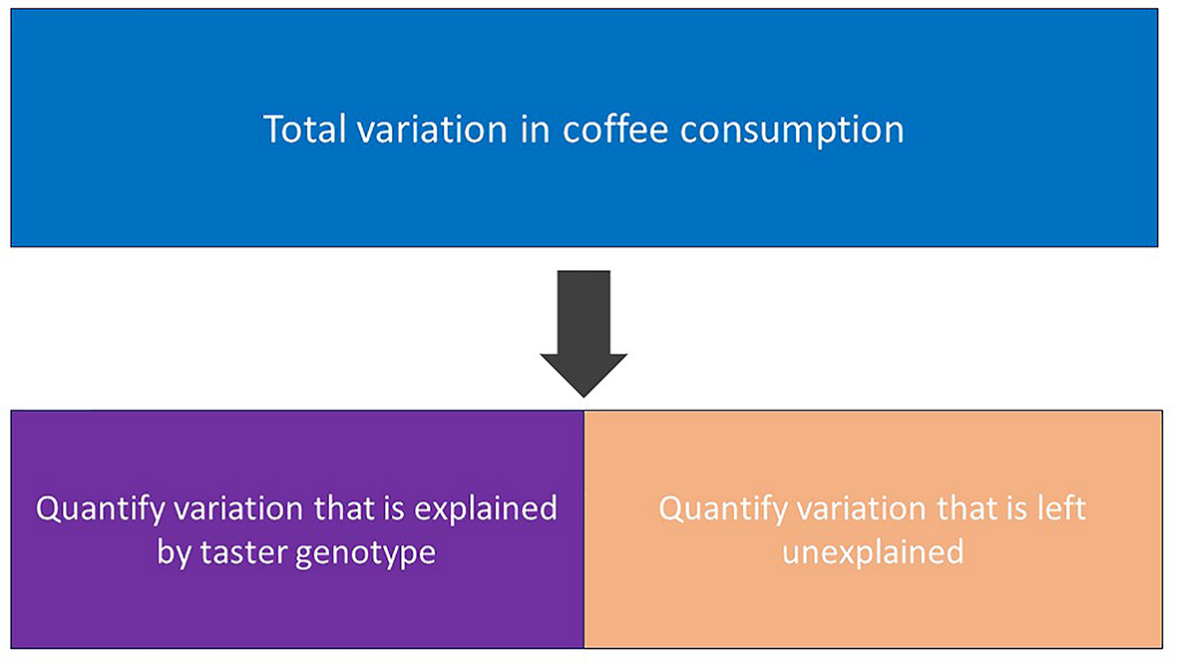
Diagram showing how total variation (sums of squares) in our response variable is partitioned between what can be explained by our explanatory variable and what is left unexplained The variation we explain can be added to the variation left unexplained to calculate total variation. This is an illustrative example and not meant to imply that exactly 50% of the variation in coffee consumption has been explained, we will only know this once we obtain the results of our GLM.

General Linear Models work by quantifying how much variation there is in our response variable, e.g. weekly coffee consumption, by calculating its sums of squares. It then partitions this total variation between what can be explained by our explanatory variable, e.g. taster genotype, and what is left unexplained, also called residual variation (Figure 11). The greater the variation explained by our explanatory variable, the better a predictor it is of our response variable and perhaps of biological importance. If the variation explained by our explanatory variable is very small then it is not really helping explain variation in our response variable and there may be less biologically important. The absolute proportion of variation explained by our model is known as R-squared (often reported as a percentage).

**Figure 12:**
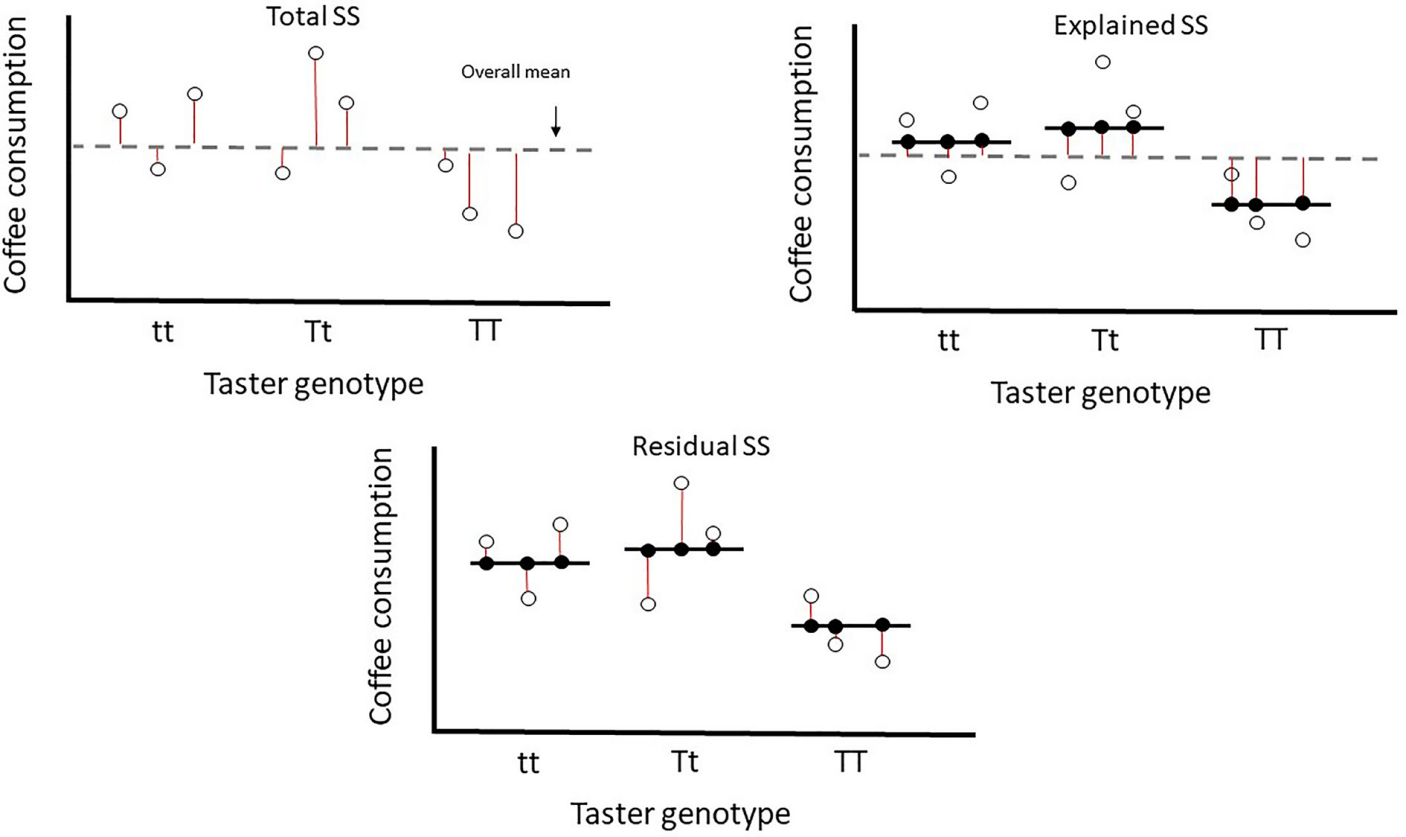
Diagram showing how total variation (sums of squares) in our response variable is partitioned between what can be explained by our explanatory variable and what is left unexplained First, the variation in our response variable is calculated by sum of squares (Total SS), the deviance for each data point from the overall mean is represented by red lines. Second, fitted values are made using our explanatory variable, the black lines show the fitted values for each group of taster genotype. The red lines represent the distance from the overall mean to our fitted values for each observation, used to calculate explained sum of squares (Explained SS or ESS). Finally, our fitted values do not explain each data point perfectly and the red lines represent the difference between our fitted and observed values. Each red line is a residual. This residual deviance is used to calculate residual sum of squares (Residual SS).

So how do GLMs actually do this. Our model will construct a linear predictor to predict our response variable, e.g. in *y* = *mx* + *c*, *y* is our response variable and ‘*mx* + *c*’ is the linear predictor with *x* being the explanatory variable. Here the ‘*m*’ would be the slope of the line of best fit and ‘*c*’ would be its intercept. That would be the case if we had a numerical explanatory variable. As our explanatory variable is a factor variable, there will a different value of ‘*x*’ for each level of our factor variable (tt, Tt, TT). We will focus on the specific linear equation of our model later, but Figure 12 demonstrates how the GLM determines the total variation in our response variable, what can be explained by our explanatory variable and what is left unexplained. This figure may be more understandable when we walk through the results of our GLM later. As with most statistics, our statistical software and a powerful computer will do these calculations for us, but it is important to understand what is being done on our behalf.

**Figure 13:**
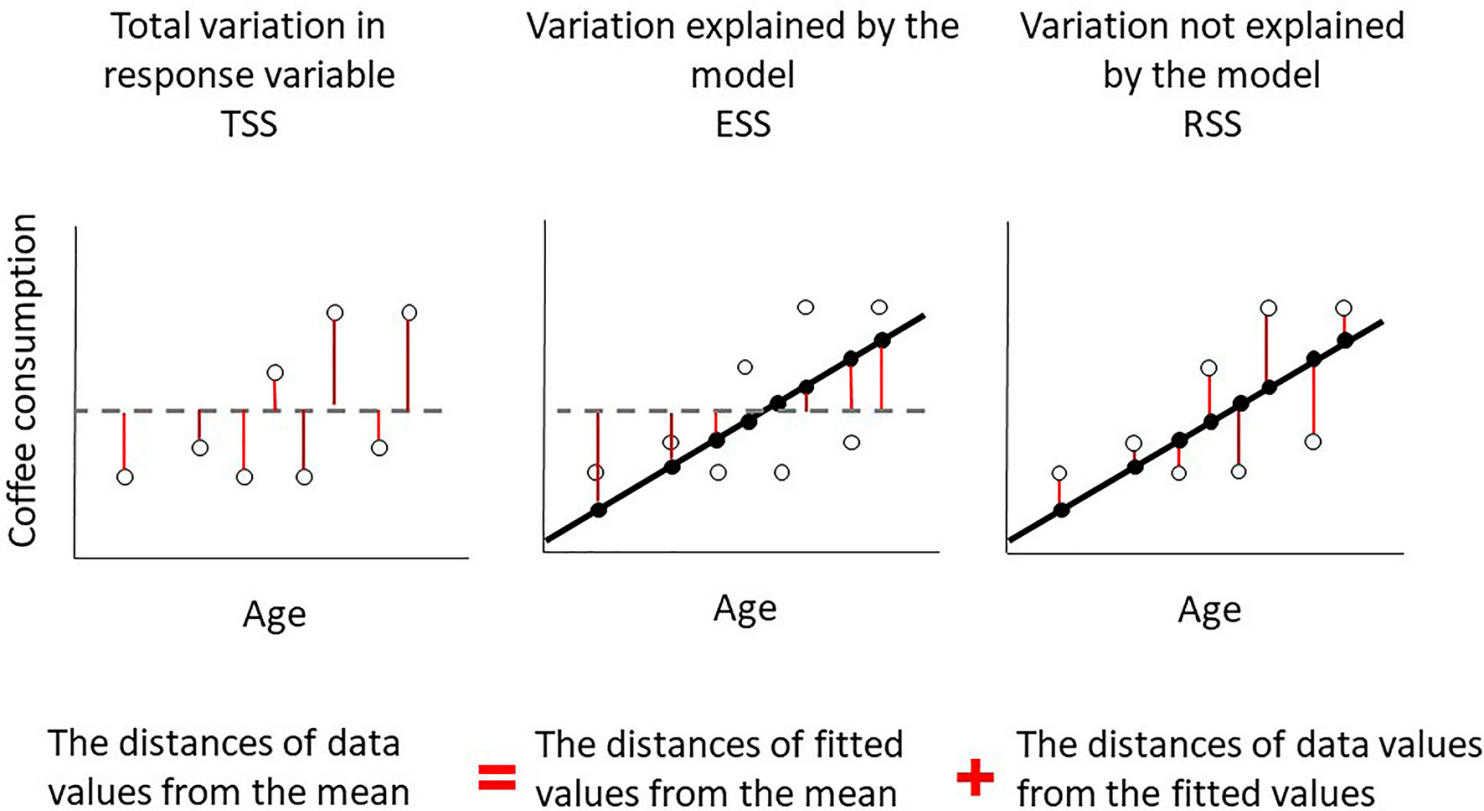
Visual illustration of how a General Linear Model works with a numerical explanatory variable (regression), i.e. quantifying whether variation in coffee consumption can be explained by age First, the variation in our response variable is calculated by sums of squares (Total SS), the deviance for each data point from the overall mean is represented by red lines. Second, a line of best fit is made using our explanatory variable, the black lines show the fitted values for each observation. The red lines represent the distance from the overall mean to our fitted values for each observation, used to calculate explained sums of squares (ESS). Finally, our fitted values do not explain each data point perfectly and the red lines represent the difference between our fitted and observed values. Each red line is a residual. This residual deviance is used to calculate residual sums of squares (Residual SS).

How would this same GLM approach work if we had a numerical explanatory variable rather than a factor explanatory variable? This would a different research question from the focus of this paper but we could have also measured the age of each of our participants and hypothesised that age explained variation in weekly coffee consumption. Figure 13 shows how the GLM will calculate a ‘line of best fit’ through our data to calculate how much variation is explained in age. As before, our model will construct a linear predictor to predict our response variable, hence why both examples are general linear models.

## Fitting a general linear model

Now we have seen how GLMs work, it is time to use them to help answer our research question. We seek to see if variation in coffee consumption can be explained by TAS2R38 genotype. First we should visualise the relationship between our variables. This is key as it gives a visual clue as to what the relationship may be and may show something unexpected about our data. As coffee consumption is a numerical variable and TAS2R38 genotype is a factor variable, we can visualise as a boxplot. We could use a barplot but that hides the variation in our data, Weissgerber et al. make an excellent case why we should be moving away from barplots and consider boxplots, violin plots or raw data plotting [4].

Figure 6 shows our boxplot. The boxplot divides each genotypes coffee consumptions into quartiles, so that each tail/whisker and each half of the box contains 25% of observations. It’s not a problem when part of a boxplot looks compressed, it means there are more observations within a small range. We can also see in the TT a circle at 20 coffees per week, this circle is an outlier. Outliers are observations that lie outside 1.5 time the Interquartile Range and essentially can be considered atypical values. They do not need to be removed from our data unless we are sure it has arisen by error.

So, what can we conclude from our boxplot? It looks like tt and Tt genotypes do not differ much in their coffee consumption but TT drink less coffee than the other groups. Perhaps being homozygous dominant (TT) for bitter taste perception makes you less likely to drink coffee due to its bitterness. This is our visual clue but we will need to fit a GLM to our data to have evidence of a relationship.

## Fitting models and interpretation of model outputs

When carrying out, or fitting, a GLM in R Studio, we will use the following command:

model1=lm(y∼x)

The ‘model1’ part of our command is the name we decide to call our model, ‘lm’ is the command to fit a linear model and we need to specify our ‘y’ and ‘x’ variables. Specifically, with our dataset named ‘PTC’ and variables named ‘Genotype’ and ‘Coffee’, we would use the following command in R:

model1=lm(Coffee∼Genotype, data=PTC)

To specify the ‘y’ variable, we name the dataset and the variable. The same is true to specify the ‘x’ variable. R will fit our GLM but we will have to use further commands to see the results.

You can obtain the ANOVA table, which shows the explained and residual variation in our response variable and test statistic, for your model in R using the following command:

anova(model1)

**Figure 14:**
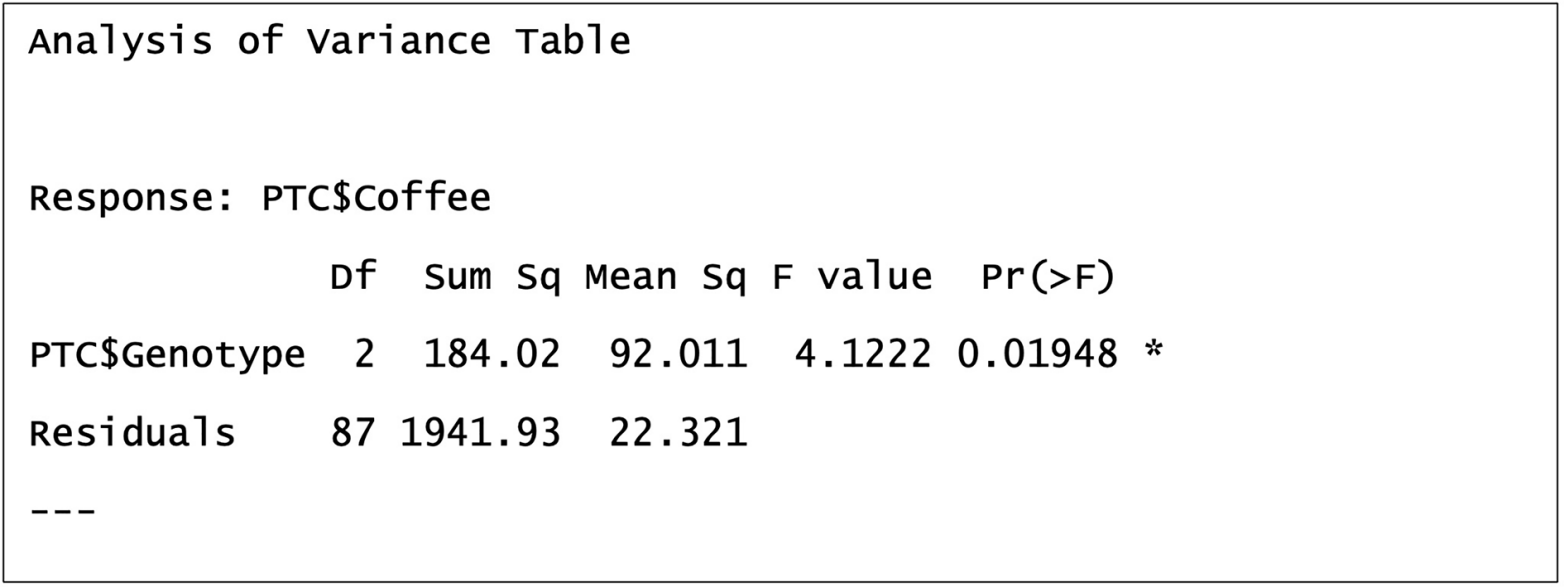
The ANOVA table from our General Linear Model

As you can see in Figure 14, we are given lots of information about our model. First we can see that the response variable of coffee consumption is stated, we have a first row of numbers relating to our explanatory variable Genotype and a second row of numbers relating to residuals. Let’s take each column in turn.

**Figure 15:**
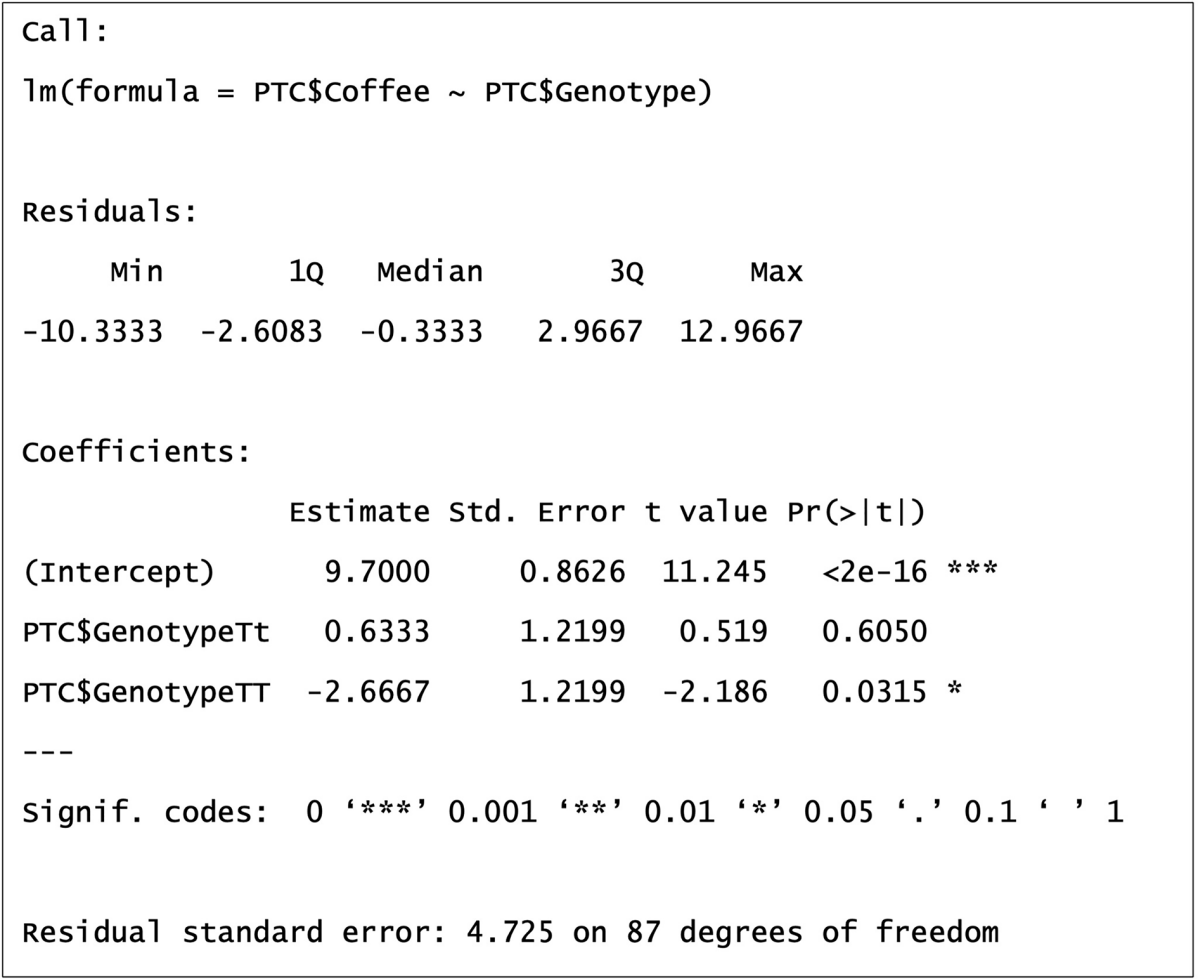
The summary output of our General Linear Model

‘Df’ stands for degrees of freedom. Two degrees of freedom have been used by our explanatory variable Genotype and 87 are residual (left over). This gives 89 total degrees of freedom, if we add 1 to this number we arrive at our sample size of 90. This is useful when degrees of freedom are reported in results as the reader can deduce the sample size of the experiment. Degrees of freedom are unique pieces of information which we use to quantify variation. The number of degrees of freedom is the number of values in the final calculation of a statistic that are free to vary. For example, if the mean of three values is 7, we know that the values must sum to 21, and if the first two values are 5 and 6, the third value must be 10. The third value has no freedom to vary. Explanatory factors always use number of levels minus 1 df and numerical explanatory variables add 1. (see Figure 15 and associated text for why this is). Explanatory numerical variables use a degree of freedom to estimate the slope (gradient) of the line of best fit (e.g. Figure 13).

‘Sum sq’ stands for sums of squares (SS), the measure of variation we calculated earlier. We can see that 184.02 SS have been explained and 1941.93 have not been explained by Genotype. We can calculate the total SS for our response variable by adding these together to get 2125.95 and calculate a multiple R-squared of 8.66% [(184.02/2125.95)*100].

‘Mean Sq’ stands for mean sums of squares (MSS) and is a standardised measure of what variation has been explained and unexplained in our response variable. This is achieved by dividing SS by the degrees of freedom used, i.e. 184.02/2 = 92.011. This is to standardise the variation we explain by the number of degrees of freedom used, so that MSS will be higher if we are efficient in our use of degrees of freedom to explain variation but lower is we use many degrees of freedom.

‘F value’ is our test statistic. It is also called the F-Ratio because it is a ratio between variation explained versus unexplained after standardising by degrees of freedom. In our example you can see that it is calculated by 92.011/22.321 = 4.1222.

‘Pr(>F)’ is our *P*-value or probability. This *P*-value represents the probability of the significance statistic being that extreme or more if the null hypothesis is true (assuming that the assumptions of the test have been met). Specifically, here it represents the probability of obtaining an F-value of 4.1222 of higher if there is no relationship between coffee consumption and genotype. The probability of this is 0.01948, which is a small probability and below 0.05.

As discussed in the hypothesis testing section, when *P*<0.05 we reject the null hypothesis and would conclude that the alternate hypothesis is supported. We would conclude from our data that TAS2R38 genotype does explain variation in weekly coffee consumption. However, we have three TAS2R38 taster genotypes, strong tasters (TT), weak tasters (Tt) and non-tasters of bitter PTC. We do not have any information about specific genotypes and where the actual differences in mean weekly coffee consumption lie. So we can obtain more information from our model with the summary output.

You can obtain the summary output for your model in R using the following command:

summary(model1)

As you can see in Figure 15, we are given further information about our model. The Multiple R-squared value of 8.66% we calculated earlier is here, which is the absolute proportion of variation explained by our model. This means over 90% of variation in coffee consumption is left unexplained. Other yet to be measured explanatory variables will help explain more of this. We may not be really interested in pursuing other variables explaining coffee consumption, but if we were measuring a particular disease and could account for only 8.66% of its variation then that is helpful information to keep researching further. This measure of R-squared would increase, if only by a little, with any further explanatory variables added to the model (as each further explanatory variable would have its own explained sums of squares (ESS) calculated), even if they have no association with the response, which is why adjusted R-squared is calculated too. This measure of R-squared penalises for this inflationary effect of additional explanatory variables, allowing a fairer comparison between models containing different number of explanatory variables. There are arguably better ways to compare models using a likelihood based approach that you may have heard of, e.g. AIC or Likelihood Ratio Tests.

The coefficient estimates are of particular interest to us and these give us some information about the mean weekly coffee consumption of separate genotype groups. Remember the model is fitting a linear equation, e.g. *y* = *mx* + *c*, and these coefficients give us values to solve our equation (explained later in Figure 17). Our first coefficient estimate of 9.7 is for the intercept (or ‘*c*’ in our linear equation), our second of 0.6333 for Tt genotype and third of −2.6667 for TT genotype. You may be wondering why is there no coefficient for tt? That is because in R the first level of a factor (alphabetically or numerically) is treated as a baseline in a GLM and given a coefficient value of 0. It is the genotype to which other genotypes are compared in their mean coffee consumption.

**Figure 16:**
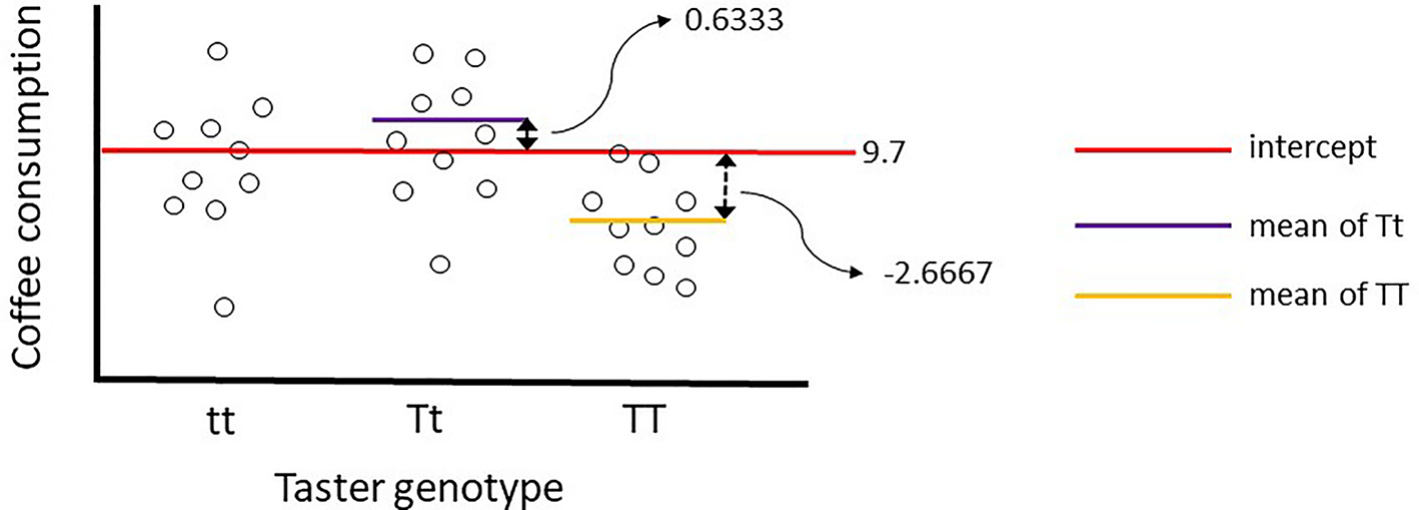
Illustrative plot of how coefficients are estimated by the GLM The numerical coefficients shown have been taken directly from the summary output of the model shown in Figure 15.

Figure 16 gives insight into how these coefficients are estimated. The intercept of 9.7 cups of coffee per week is also the mean coffee consumption of the tt genotype group. To arrive at the mean weekly coffee consumption for the Tt group we need to add 0.6333 to the intercept, and for the TT group we add --2.6667.

**Figure 17:**
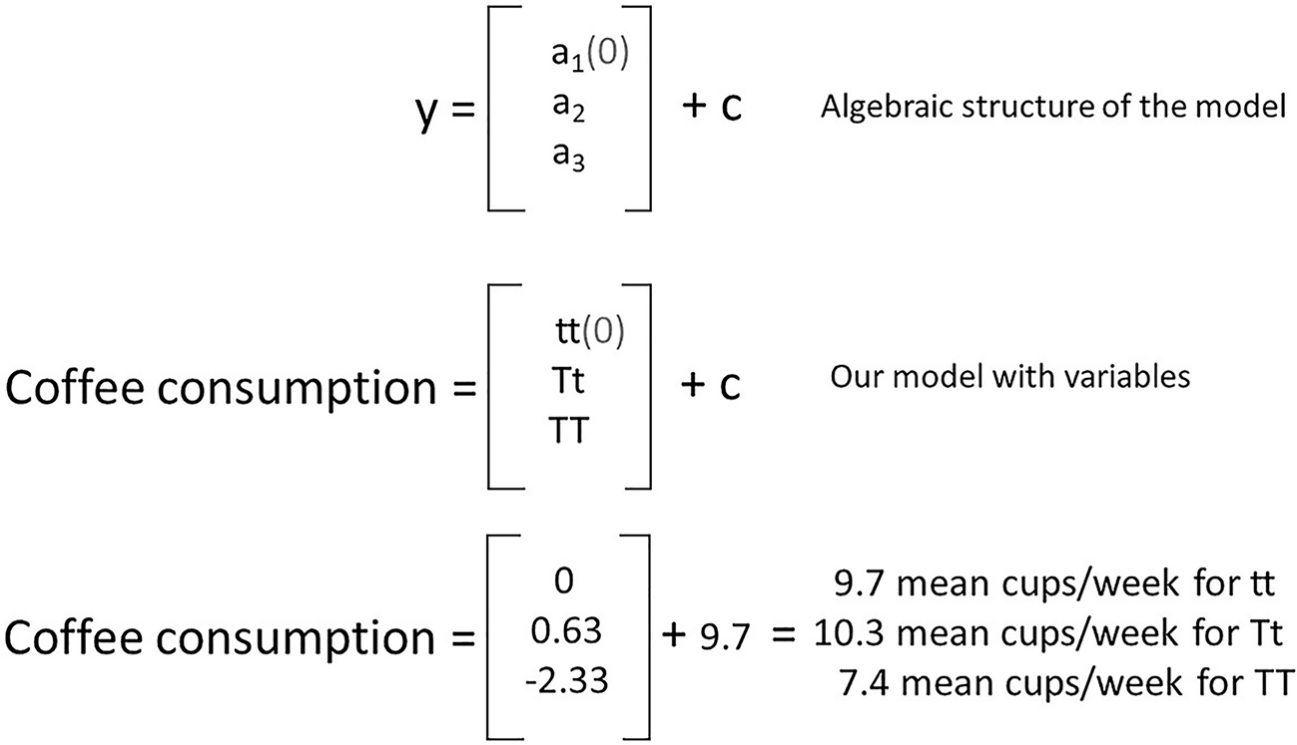
The structure of our model showing the linear equation and replacing with coefficients from our model to predict mean weekly coffee consumption for each TAS2R38 taster genotype The top row shows the general structure of a model with a three-level factor explanatory variable, the middle row shows the same model with the variable labels indicating the genotypes for our example, and the bottom row shows the same model with the variables replaced by the estimated coefficient values

Not only can we appreciate how GLMs work but also that they can be used to predict values of our response variable (Figure 17). We could have achieved this quickly be calculating the mean of each genotype using summary statistics as this is a simple model with one explanatory variable. The biological world is extremely complex and often we may wish to consider many explanatory variables and their interactions in a single model. With more complex models, our GLM will estimate more coefficients but still allow us to make predictions and estimate uncertainty around those predictions. This is an extremely valuable and powerful tool for us as biologists. Not only can GLMs determine where significant differences or relationships may lie but they can have predictive power.

**Figure 18:**
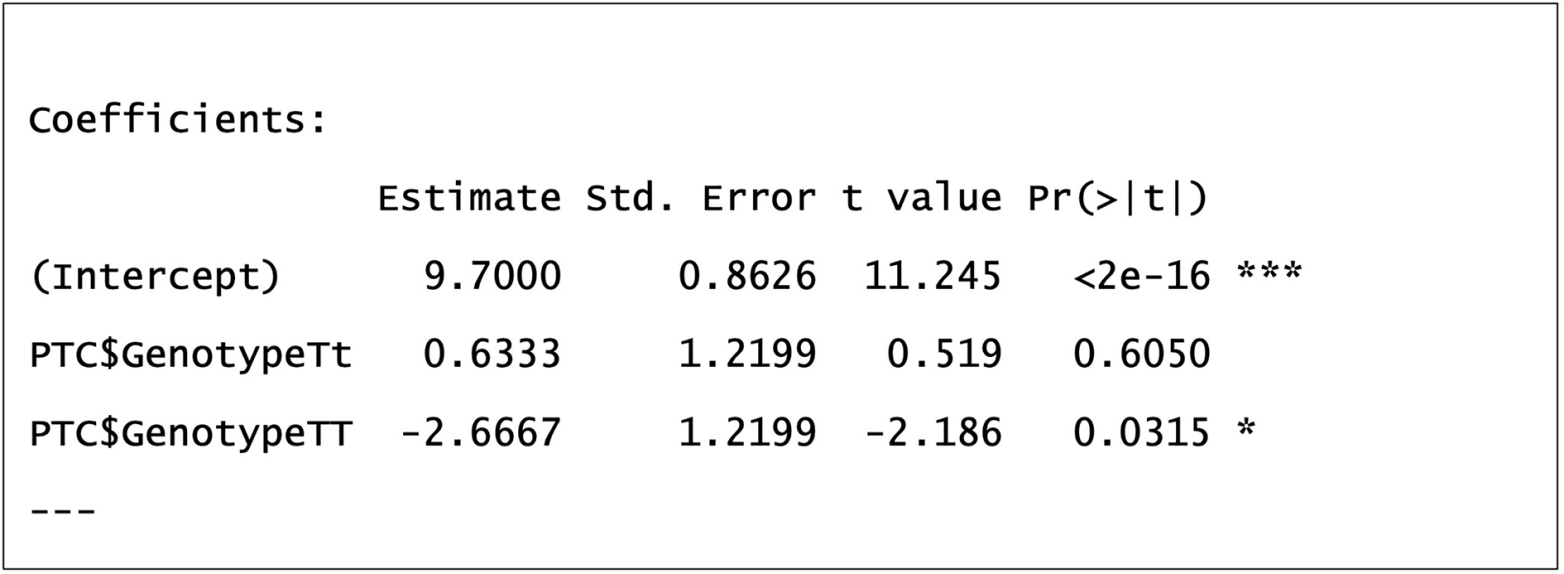
Coefficient estimates from the summary output of our GLM

Lets return to our summary output and focus on other information supplied with coefficients (Figure 18). When we estimate a coefficient, we estimate a mean and we do so with error. Standard error (SE) ‘Std.Error’ is a measure of this error and it is the standard deviation of our coefficients (i.e. the se is for the mean what the sd is for the group data). The *t*-value is the number of standard errors our coefficient is from zero, i.e. from the baseline genotype tt of 9.7. The *t*-value for Tt is 0.519, showing that the mean coffee consumption of Tt genotype 0.519 standard errors above the tt genotype. If we move 0.519 standard deviations away from a baseline mean of a normal distribution, we are still very much within the bell-curve of that distribution. This is supported by our corresponding p-value of 0.605. There is no significant difference between the mean weekly coffee consumptions of tt and Tt genotypes. The *t*-value for TT is -2.186, showing that the mean coffee consumption of Tt genotype 2.186 standard errors below the tt genotype. If we move 2.186 standard deviations away from a baseline mean of a normal distribution, we are outside almost all of the bell-curve of that distribution and perhaps significantly different from it. This is supported by our corresponding *P*-value of 0.03. We can conclude mean weekly coffee consumptions of the TT genotype group is less than the tt genotype group. There is one comparison missing, Tt v TT, but as these are the highest and lowest mean coffee consuming groups respectively, we can deduce that they are significantly different from each other. To be sure we could re-run our model and change the baseline genotype group. Alternatively we could perform a post-hoc test such as a Tukey test, which tests whichgroup means are significantly different from other group means. This involves multiple pairwise comparisons and performing multiple tests increase the chance of a false positive result (Type I error), which is why the Tukey test provides adjusted *P*-values in an attempt to mitigate this risk.

## Reporting our results

Sharing our findings with others is a key skill and part of the importance of research. Clarity and authenticity are key. We have the results of our model and understand how they were derived and what they mean. Reporting of statistical results is achieved by an accessible description of the finding supported by evidence from your model. Our model has provided us with substantial information but it is a common practice to report three of these: the test statistic, degrees of freedom and *P*-value. For example, we may report the results from the ANOVA table as:

‘Variation in coffee consumption (cups per week) was explained by TAS2R38 taster genotype (F_2,87_ = 4.122, *P*=0.019)’

We elaborate on these with the results from the summary output with:

‘Participants with the Tt TAS2R38 taster genotype did not differ significantly in their coffee consumption from the tt group (*t* = 0.519, *P*=0.605). However, the TT group drank significantly less coffee on average than the tt group (*t* = −2.186, *P*=0.03)’

There is a lot of variety on how we describe our results, and how you may report your results may depend on your research question and where you want to focus. It may be relevant to make predictions from your model, it may be relevant to state what test was used as well. It is almost always helpful to report summary statistics, e.g. state the mean ± SD coffee consumption for each genotype. Consider what is an effective and authentic portrayal of your results. It is almost always appropriate to accompany your model with a plot, so that you can visualise the relationship that you are modelling.

## Conclusions and limitations

We started with a research question and a sample of data to help answer it. We explored how we can understand and describe our data with summary statistics and use a GLM to ultimately answer our research question. This approach can be used to answer research questions across biological disciplines and beyond, so is extremely helpful. This has been achieved with a sample of data, which as discussed may not be sufficiently representative to make confident conclusions about the wider population. However key potential limitations or even errors in our results can be due to our choice of statistical test and model. These are key to check so we can have greater confidence in our conclusions.

## Model assumptions/diagnostics

Unfortunately the power and versality of GLMs comes with a cost. There are assumptions associated with statistical tests and we need to check these to ensure our model is reliable. It would be problematic if we concluded the wrong result to our research question because the assumptions of a test were not met. GLMs are fairly robust and small deviations from these assumptions are often not too detrimental. However, the biological research questions we ask are serious and it is key to consider whether the model we have fitted is suitable to make predictions and conclusions, or if another model is better suited.

There are three key assumptions that have to be met for fitting GLMs. This is not an exhaustive examination into these assumptions but we aim to equip you in how to check them.

### Residuals are normally distributed

**Figure 19:**
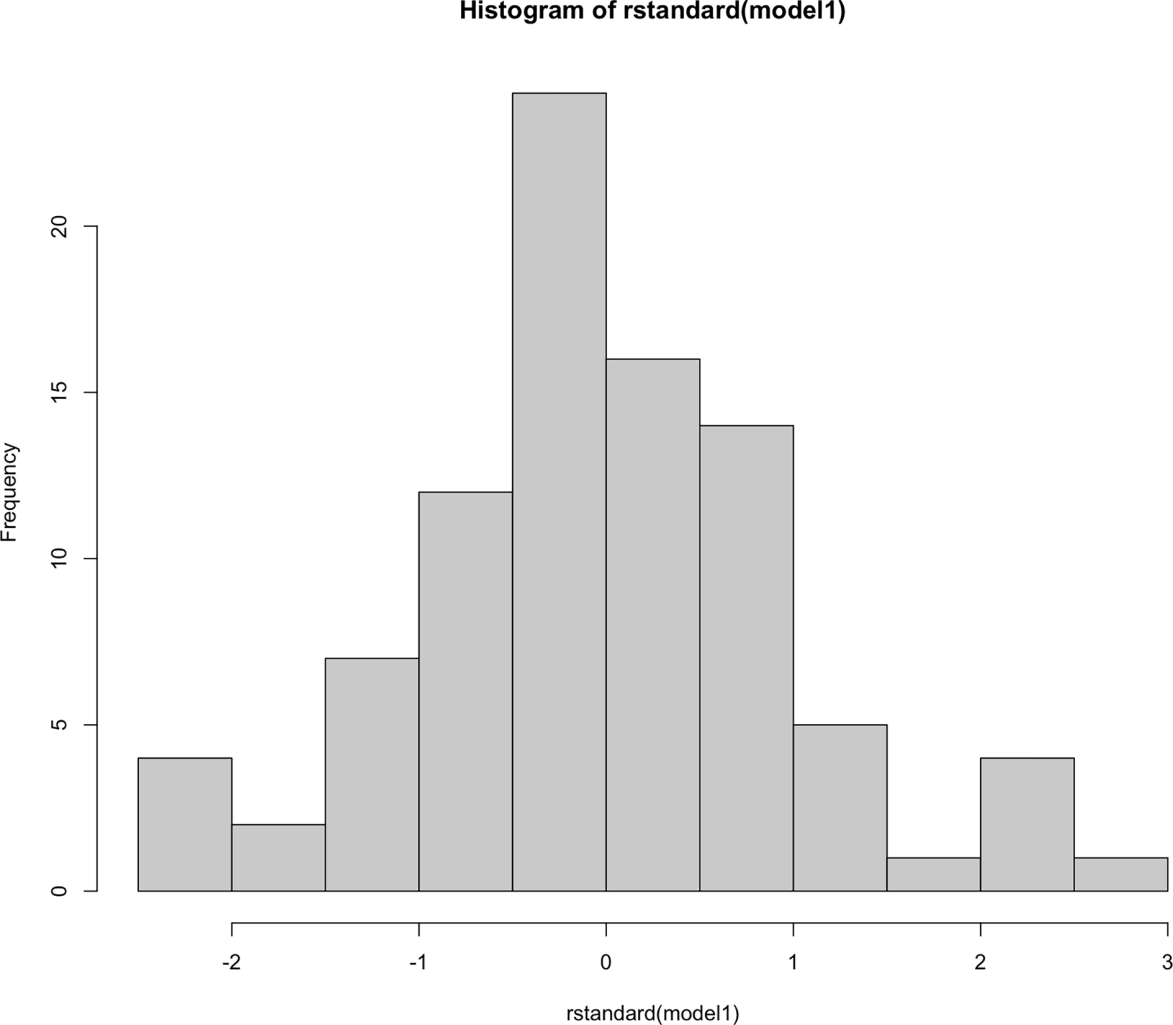
Histogram of the residuals from our General Linear Model They should be normally distributed and appear similar to a bell-shaped curve. Our residuals are not perfectly normally distributed, but they are fairly evenly spread around a central peak at zero and we could conclude they are fairly normally distributed.

An important point, it is not our response variable that needs to be normally distributed, but, rather, the residuals need to be normally distributed. Our residuals (represented by our red lines in the final parts of Figures 11 and 12) are the distances between our observations and our fitted model. Some observations are very close to the model fit and will have a small value, others may be much higher than the fitted model and have a large positive value. We can check these via either a histogram of residuals (Figure 19) or a Q-Q plot (explained subsequently). You can check with formal tests, such as Shapiro–Wilk, but these can be influenced substantially by sample size. Statistical power is the probability a hypothesis test can detect an effect in a sample when it exists in the population. With a very large dataset and therefore high statistical power you will detect very small deviations from normality when the residuals visually appear appropriate.

### Residuals are independent of each other

If our residuals are not independent of each other, it means they are connected in some way and therefore the observations are connected. Consider if there is anyway that knowing something about one residual informs you about another? For example in our research question, we may have sampled multiple individuals from the same family or who live together and as a consequence share dietary preferences and have similar coffee consumption. Another example is if we sampled the same individual multiple times by accident. Sometimes this problem can be avoided by good experimental design or by including that variable in the model, e.g. family as a random effect. If you are analysing data already collected, then you do not have control over the experimental design but knowledge of how the data were collected can highlight the aforementioned problems. It can also be detected by plotting residuals in the order they appear in your dataset or by spatial variables in your dataset, any patterns can suggest non-independence.

### Homoscedasticity

Not the easiest word to pronounce in statistics but it represents the consistency of data spread around the model fit. It assumes equal or similar variances in different groups being compared. In a GLM, we expect the observations to be relatively consistently spread around the model fit. If this is not the case, the issue is that we are making much more accurate predictions of *y* at certain values of *x* and less accurate predictions at other values of *x*. This could be resolved by a transformation of one or both axes. Alternatively more complex models may help here. One quick way to check this is by looking at our original boxplot (Figure 6), if we have fairly constant variation across each level of our factor explanatory variable then each box would be similar in size. Perhaps the TT boxplot shows a little less variation than other genotypes but it is definitely not extremely different.

**Figure 20:**
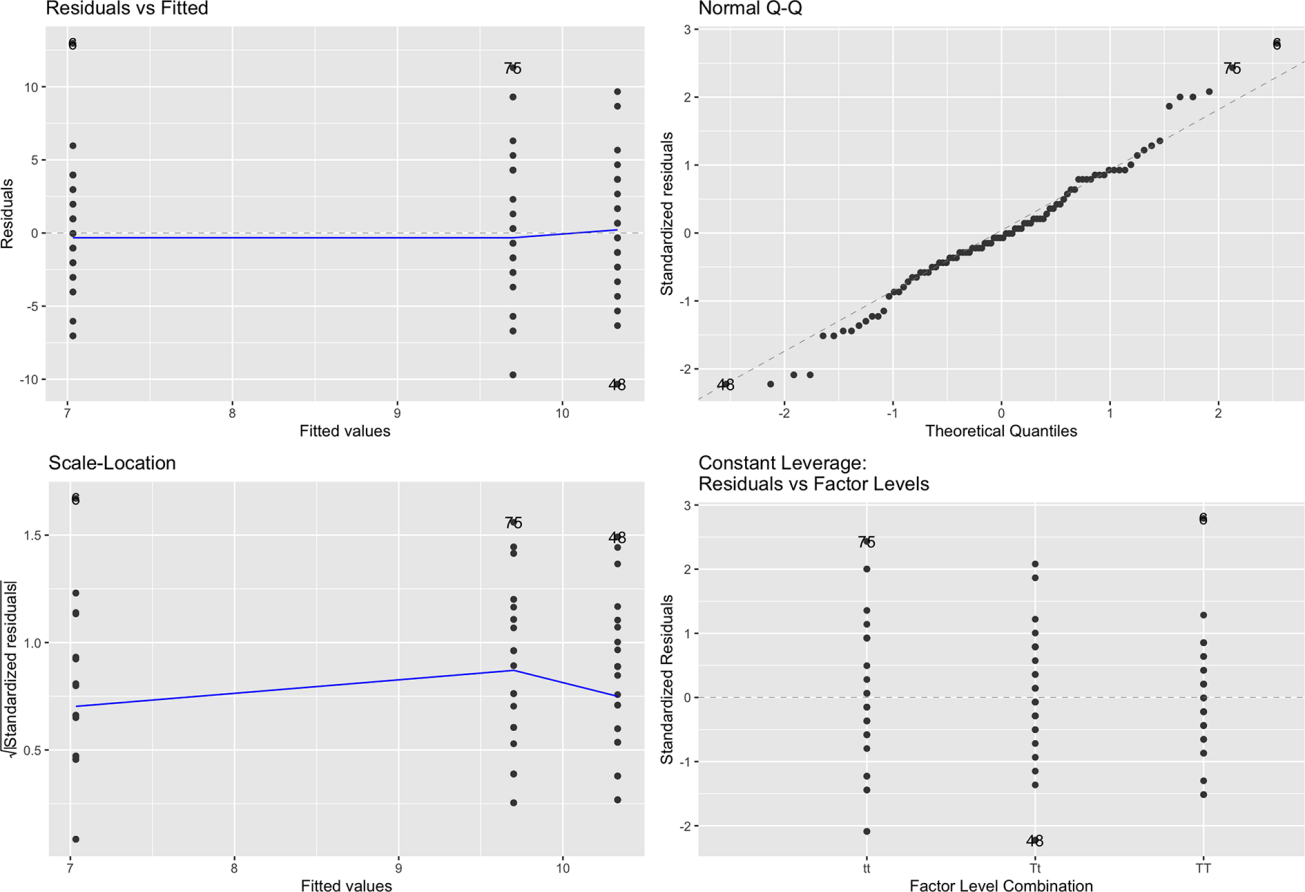
Autoplot of our General Linear Model from the R package ‘ggfortify’

A really quick and useful way to check your model diagnostics is with the autoplot function in the ggfortify package of R (there are similar plots in other statistical software). This will produce 4 plots that check different aspects of your model (Figure 20).

The first plot in our model diagnostics is called ‘Residuals vs Fitted’. This is to detect non-linearity and if the blue fitted line is horizontal then we can be satisfied that the linear equation we have used to describe our data is ok. We may see the blue line following a large curve or hump if the model was not suitable.

The Q–Q plot informs us about the distribution of our residuals, as does the histogram in Figure 19. The black data points are our actual residuals and the dashed line where they would be expected under a normal distribution. The vast majority of our residuals fall on the dashed line and some of the outliers that don’t are labelled, e.g. ‘76’ is the residual from the 76th observation in our dataset (this person drank a lot more coffee than others in their genotype group, which may be of interest to us). Overall we can be satisfied here.

The scale-location plot informs us about homoscedasticity. Our black data points appear in three columns as we have fitted values for three genotypes. Again we are expecting a reasonably horizontal blue line and that is what we have here. This confirms what we already saw in our boxplot, we have fairly constant variation across each level of our factor explanatory variable.

The constant leverage plot is helpful to detect whether there are influential datapoints influencing our results, for example an outlier. Our black data points here are fairly evenly spread across each level of our factor explanatory variable. A red line may also appear on this plot which we hope is fairly horizontal but would appear heavily skewed by influential datapoints that may be influencing our overall result. Imagine one of our participants was the world coffee drinking champion, they would skew the mean coffee consumption in their genotype group up and may affect the overall result of the study. This would be picked up by this plot.

A further diagnostic check that is particularly important to consider in biology is that of collinearity between explanatory variables. Collinearity is when we have two (or more) variables that are correlated. When fitting GLMs with multiple explanatory variables, there will likely be some overlap in the variation of the response variable explained by each explanatory variable. If our explanatory variables overlap too much, it may appear that the first explanatory variable explains a significant variation but the second does not, purely as a consequence of the order they are considered in the model. For example, we may seek to explain Type 2 diabetes incidence with the explanatory variables of participant weight and Body Mass Index (BMI) and find that weight masks the effect of BMI on diabetes in the model output. This is because BMI is calculated from body weight, a high weight will often correspond to a high BMI, so this is why both variables may overlap in the variation they explain. We would not wish to incorrectly conclude that BMI is not related to Type 2 diabetes incidence purely due to the order they are considered by the model. Collinearity is not a problem if it is identified. There are multiple ways to check and evaluate, with a simple check being to re-run your model with the order of explanatory variables reversed. If the results stay the same, then collinearity isn't negatively influencing you results and conclusions.

## What may cause model assumptions not to be met and what else could we do?

A small sample size may often lead to model assumptions not being met. Even if sampling from a normal distribution, a small number of observations may appear quite different from this. A larger sample size likely be more representative of the phenomenon we are trying to understand.

Other common reasons can arise from the nature of the data we are collecting. If our response variable is a count with a low mean (e.g. counts of 0, 1, 2, and 3 are common), then our diagnostics will likely not be met. If our response variable is binary with yes/no or 0/1 outcomes, then our diagnostics will likely not be met. In biological research, counts or binary response variables are common. The point here is that we are no longer sampling from a normal distribution (defined by a mean and standard deviation), how could we if we can only observe 0 and 1 values. Understanding this help us model our response variable with a more appropriate distribution, e.g. Poisson distribution for count data and Bernoulli distribution for binary data. These are called Generalised Linear Models, which as the name suggests are quite similar to General Linear Models. These tests will also estimate coefficients and therefore are parametric tests. We won’t cover them here but would like to point out that the GLMs we have covered in this article are a solid foundation to analysing other types of data. This is why we advocate a linear modelling approach, as it could suit most of your biostatistics needs.

Transformations of one or more variables can potentially address failed diagnostics. It could be that your GLM of coffee∼genotype fails diagnostics but (log)coffee∼genotype does not. The popularity of this approach can vary across research areas, e.g. mass spectrometry data are often log-normal and log-transformed for analysis. Coefficients can still be estimated but we will have to reverse transform in the process of making predictions. In this example, (log)coffee∼genotype would be the more appropriate GLM but it might be more difficult for us to interpret what the biological meaning of (log)coffee consumption is.

There are also non-parametric approaches as a potential alternative when assumptions of parametric tests are not met. GLMs are examples of parametric test because we are estimating parameters with our coefficients, and therefore can conclude that the TT group drank 2.67 cups less coffee on average than the tt group, which was significant. Non-parametric tests often rank our data for analysis, so our largest coffee drinker is ranked 1st rather than being a 21 cups per week drinker. Therefore this approach can potentially detect whether the tt group drinks more coffee that TT but does not estimate differences between groups via coefficients. Specifically in our research question we could have used a Kruskal–Wallis test instead. Non-parametric approaches do not estimate parameters (coefficients), so they cannot be used to make predictions of our response variable. Non-parametric tests are distribution free and not influenced by outliers. This is because they rank our observations for analysis, so could detect which genotype group contained more high coffee consuming individuals and still provide us with an answer to our research question. However, we would need to calculate means and variation within genotypes separately.

## Closing remarks

As biologists we seek to answer research questions. Most research questions can be asked in a univariate framework, that is have a single response variable that we want to explain variation in. Summary statistics will help us describe the variation in our response and explanatory variables. Data visualisation will help us visualise potential relationships or unexpected observations. General Linear Models help us explain variation in our response variable and arrive at conclusions. Like all tests, they have assumptions we should check and appreciate.

This is a process, applying statistics to answer research questions. Practice will help you grow in experience and understand more about data analysis. Ultimately this will help us to arrive at answers to our research questions in a reliable and robust way.

## Summary

- As biologists we need to use statistics to help answer biological research questions.
- This article will cover how we can apply descriptive statistics, data visualisation and distributions, probability, hypothesis testing, General Linear Models and model assumptions/diagnostics.
- This statistical approach for biologists is covered utilising an accessible biological research question throughout, using the popular statistical programming language R.

## Supplementary Material

Supplementary Material

## Abbreviations

ANOVA: analysis of variance
BMI: Body Mass Index
ESS: explained sums of squares
GLM: General Linear Model
PTC: phenylthiocarbamide
SD: standard deviation
SE: standard error
